# Simulating variance heterogeneity in quantitative genome wide association studies

**DOI:** 10.1186/s12859-018-2061-1

**Published:** 2018-03-21

**Authors:** Ahmad Al Kawam, Mustafa Alshawaqfeh, James J. Cai, Erchin Serpedin, Aniruddha Datta

**Affiliations:** 1 Electrical & Computer Engineering Dept., Texas A&M University, College Station, TX, USA; 2 Veterinary Integrative Biosciences Dept., Texas A&M University, College Station, TX, USA; 3 TEES AgriLife Center for Bioinformatics and Genomic Systems Engineering (CBGSE), Texas A&M University, College Station, TX, USA; 4 Electrical & Computer Engineering Dept., German Jordanian University, Amman, Jordan

**Keywords:** Variance heterogeneity, Genome wide association studies, GWAS simulation

## Abstract

**Background:**

Analyzing Variance heterogeneity in genome wide association studies (vGWAS) is an emerging approach for detecting genetic loci involved in gene-gene and gene-environment interactions. vGWAS analysis detects variability in phenotype values across genotypes, as opposed to typical GWAS analysis, which detects variations in the mean phenotype value.

**Results:**

A handful of vGWAS analysis methods have been recently introduced in the literature. However, very little work has been done for evaluating these methods. To enable the development of better vGWAS analysis methods, this work presents the first quantitative vGWAS simulation procedure. To that end, we describe the mathematical framework and algorithm for generating quantitative vGWAS phenotype data from genotype profiles. Our simulation model accounts for both haploid and diploid genotypes under different modes of dominance. Our model is also able to simulate any number of genetic loci causing mean and variance heterogeneity.

**Conclusions:**

We demonstrate the utility of our simulation procedure through generating a variety of genetic loci types to evaluate common GWAS and vGWAS analysis methods. The results of this evaluation highlight the challenges current tools face in detecting GWAS and vGWAS loci.

## Background

Genome-wide association studies (GWAS) have been widely utilized over the past decade for studying the genetic origins of phenotypic traits, including a wide range of diseases. GWAS analysis compares the genome-wide genotype profiles of a large number of subjects with the corresponding phenotypic values. These studies aim to locate genetic loci where the genotype variation is highly correlated with the variation of the phenotype value [1–3]. These studies, however, have been limited to studying variation in the mean phenotype value. That is, the mean phenotype value observed when a certain genotype is present will be significantly different from the mean phenotype value observed when an alternative genotype is present. Although this type of analysis has been successful in uncovering a wide range of genetic associations, detecting mean changes only explains a small part of the total variance [4]. The remaining variance has been thought to be caused by gene-gene (GxG) interactions, such as regulatory factors and epistasis, and gene-environment (GxE) interactions. These types of interactions cannot be easily detected using typical GWAS methods.

To address the limitations of typical GWAS analysis methods, vGWAS has emerged recently as an approach for detecting GxG and GxE loci [5–7]. There has been increasing evidence that when GxG or GxE interactions occur, variance heterogeneity is introduced into the genetic locus at which the interactions occur [8]. Therefore, detecting vGWAS loci enables researchers to identify and study genetic loci involved in these interactions. vGWAS analysis aim to detect genetic loci that cause a significant change in the variance of the phenotype value. That is, the variance of the phenotype value observed when a certain genotype is present will be significantly different from the variance value observed when an alternative genotype is present.

The number of vGWAS analysis methods have been steadily increasing in the literature in the past few years. These methods utilize statistical tests and regression models to locate loci that have statistically significant effects on the phenotype. For example, Levene’s (Brown-Forsythe) test is a single-pass statistic widely used in vGWAS analysis. The test calculates the phenotype’s variance for both values of the genotype and then assesses the significance of the difference between the two variances using a chi-distribution. Generalized linear models have also been proposed for vGWAS analysis. These methods aim to fit a linear regression model to uncover relations between the genotype profiles and the observed phenotype values. In these models, the genotypes represent the variables of the model, whereas their coefficients represent their contribution to the mean and variance changes [6]. As opposed to typical GWAS analysis linear models, vGWAS models assume that the dispersion of the model is a function of the genotype values. The authors of [9] used a double-generalized linear model (DGLM) to account for population specific effects in addition to mean and variance heterogeneity.

These methods have been utilized to uncover GxG and GxE interacting loci in a number of studies. For example in a study about body mass, vGWAS identified a locus that is believed to interact with physical activity and the lifestyle of the individual [10]. In another study of the human genome, most of the vGWAS loci identified were located in CNV-containing regions, suggesting a link between variance heterogeneity and the effects of CNVs which include having a higher susceptibility to various complex neurological disorders such as schizophrenia and Parkinson disease [9]. Finally, the vGWAS analysis of lymphoblastoid cell lines identified two genetic loci which have been confirmed to be involved in GxE and GxG interactions [11].

The proposed methods have been able to uncover biologically relevant information through detecting variance heterogeneity in different GWAS datasets. However, the development of vGWAS analysis models could still benefit from significant enhancements. A first step towards achieving that goal could be establishing reliable and consistent grounds for comparison between these tools. This could be achieved through simulating vGWAS loci. Simulation provides the researcher with three important features: 1) Control over the input genotypes, 2) Predictability of the output values, and 3) Consistency across different runs. Controlling the values of the input genotypes allows the researcher to simulate different genetic and evolutionary scenarios. The predictability of the output is particularly valuable for evaluating vGWAS methods as it provides a ground truth for comparison. And finally, consistency is achieved through eliminating the uncontrollable factors of randomness that are usually present in lab experiments. Therefore, simulation presents a reliable and inexpensive method for testing and evaluating vGWAS methods under different conditions.

Several GWAS simulators have been developed in the past decade, however, none of these simulators account for variance heterogeneity effects. In this work we present a systematic procedure for simulating vGWAS data, as well as introducing the first simulation algorithm for quantitative vGWAS data. The algorithm could be applied to either haploid or diploid genotypes with different modes of dominance while incorporating the effects of multiple vGWAS loci using an additive model. We hope that the presented work will motivate the evaluation of current vGWAS methods and drive the development of new methods.

This paper is organized as follows: we describe the genotype generation process and the subsequent mathematical framework for producing vGWAS phenotype values in the “Methods” section. We then describe the implementation of our vGWAS simulation algorithm and demonstrate it’s utility in the “Results and discussion” section before presenting our conclusions in the last section. We present a detailed mathematical discussion in the Supplementary Materials section.

## Methods

The simulation of GWAS data enables researchers to investigate important aspects of genetic control [12]. Simulation grants researchers control over the genetic properties of the input population and allows them to observe the impact of using different genetic models on the output. The main contribution of GWAS simulation is that it provides a tool for evaluating the statistical and algorithmic methods developed for detecting genetic control loci. Simulation allows the researcher to set a ground truth for these methods to be compared against, as well as enabling the evaluation of these methods under different scenarios.

The simulation process is divided into two steps. The first step of the simulation process focuses on producing a realistic population of genotypes while allowing control over several genetic and evolutionary parameters. The second step of the simulation process assigns or calculates phenotypic values for the simulated population. In this section, we describe both steps of the simulation process.

### Population generation

The population generation process focuses on producing a realistic population of genotypes. Population generation is used to simulate different genetic properties and population structures, such as demographies, mutations, and recombination events. This level of control allows the researcher to engineer the population according to the genetic and evolutionary scenarios investigated. Several simulation methods have been developed to address this problem. These methods could be categories into three types, depending on the simulation strategy they use: resampling-based methods [13–15], backward- time-based (coalescent) methods [16–18], and forward-time-based methods [12, 19–22].Each of these strategies provides the researcher with varying levels of control over the different aspects of the simulation. However, the vGWAS simulation method we describe in this paper could be applied to genotypes from any type of population simulator.

### Phenotype assignment

Phenotype assignment maps the genotype state of each simulated individual to a phenotype value. Phenotype values could be either quantitative or qualitative. Qualitative phenotypes are usually assigned in case/control studies where the phenotype could be one of only two values. Quantitative phenotypes on the other hand take a numerical value which could represent traits such as height, weight, or expression level of a certain gene.

In this section we formulate a method for simulating quantitative phenotypes. We discuss the steps for calculating the phenotype value for both haploid or diploid genomes using either an additive or dominant mode of inheritance in the diploid case. We extend our calculation method to incorporate effects from multiple vGWAS loci using an additive model for genetic control. To facilitate the phenotype value calculation we make the following two assumptions:

1. The individual has a haploid genome.
2. The phenotype value is affected by the allelic value of a single genetic locus.

In typical GWAS simulations, the general model used in calculating the quantitative phenotype value expresses the phenotype value as a function of the subject’s genotype state at the associated genetic loci in the presence of a normally distributed residual variance. The phenotype value calculation is performed according to Eq. 1, where *y* is the phenotype value, *μ* is the baseline mean value, *g*∈0,1 is the genotype state and *α* is the mean shift. *ε* represents the residual effects.

1 $$  y = \mu + g\alpha + \epsilon, \quad \epsilon \sim N\left(0, \sigma_{E}^{2}\right)  $$

In typical GWAS models, *ε* is assumed to follow a normal distribution with a fixed variance $ \sigma _{E}^{2}$. However, in vGWAS models the variance is dissected into two parts, a part that is affected by the individual’s genotype, and a remaining residual variance due to non-genetic environmental effects [7]. Therefore, in our model *ε* is expressed as shown in Eq. 2, where *σ* is the baseline standard deviation, *g* is the genotype state, and *ϕ* is the standard deviation shift due to the minor allele.

2 $$  \sigma_{E} = \sigma + g\phi  $$

### Phenotype calculation framework

The phenotype calculation step uses the generated genotype profiles to calculate a phenotype value for each subject in the population. The genotype profiles are used to calculate the allele frequencies of each loci. The researcher then specifies the phenotype controlling loci and the effect size of each locus on the total phenotypic variation. In typical settings, the researcher would choose specific loci from the list of generated loci. Alternatively, the researcher could specify the number of phenotype controlling loci and an allele frequency range such that the simulator could pick appropriate loci from the list. The phenotype calculation process is often provided with parameters such as a baseline mean and the value of the total phenotype variation in order to control the range of possible phenotype values. This allows the generated phenotype values to resemble the values encountered in the researcher’s investigation. In this section, we show how these inputs could be used to calculate the parameters of Eq. 1 and therefore calculate the corresponding phenotype value.

Based on the two assumptions made earlier, each locus could have either one of two genotype values. The characteristics of each genotype can be summarized in Table 1, where *A*_1_ is the major frequency allele and *A*_2_ is the minor frequency allele. The probability of observing a major frequency allele is denoted by *p*. Similarly *q* is the probability of observing a minor frequency allele.

3 $$  V_{Y} = V_{M} + V_{V} + V_{R}  $$

**Table 1:** Genotype Characteristics

Genotype	Frequency	Mean	S.D.
*A*_1_	*p*	*μ*	*σ*
*A*_2_	*q*=1−*p*	*μ*+*α*	*σ*+*ϕ*

According to the vGWAS model, the phenotype calculated using Eq. 1 has three sources of variation: As shown in Eq. 3, the total phenotype variance *V*_*Y*_ is composed of the addition of the variance due to the mean shift, *V*_*M*_, the variance due to the variance shift between genotypes, *V*_*V*_, and the remaining residual variance *V*_*R*_. We translate to mathematical terms in Eq. 4 to obtain the expression for each type of variance.

4 $$  \begin{aligned} Var(Y) &= Var(E[Y|G]) + E[Var(Y|G)] \\ &= Var(\mu+g\alpha) + Var(\sigma+g\phi) + E^{2}[\sigma+g\phi] \\ \end{aligned}  $$

By comparing Eq. 3 with Eq. 4 we can map each type of variance to its corresponding mathematical term. This allows us to expand these mathematical terms to reach an expression that relates each type variance to the allele frequency at the locus, as well as to the unknown parameters of the phenotype calculation process, as demonstrated in Eqs. (5) to (7).

5 $$  \begin{aligned} V_{M} &= Var(\mu+g\alpha)\\ &= pq\alpha^{2}\\ \end{aligned}  $$

and,

6 $$  \begin{aligned} V_{V} &= Var(\sigma+g\phi)\\ &= pq\phi^{2}\\ \end{aligned}  $$

Consequently,

7 $$  V_{R} = E^{2}[\sigma+g\phi] = (\sigma+q\phi)^{2}\\  $$

The locus’s effect size on the total variance due to the mean shift, *C*_*μ*_, is calculated as *C*_*μ*_=*V*_*M*_/*V*_*Y*_. Similarly, the locus’s effect size on the total variance due to the variance shift, *C*_*V*_, is calculated as *C*_*V*_=*V*_*V*_/*V*_*Y*_. Following these two terms, we can reach a formulation that expresses *α*, *ϕ*, and *σ* in terms of the total variance, the effect size of the locus, and the corresponding allele frequency, all of which are known at simulation time. These expressions are given in Eqs. (8) to (10).

8 $$  \alpha = \sqrt{C_{\mu} V_{Y} / pq}  $$

9 $$  \phi = \sqrt{C_{V} V_{Y} / pq}  $$

The phenotype value for every individual in the population is calculated through substituting the above terms in Eq. 1.

10 $$  \sigma = \sqrt{V_{Y}(1-(C_{\mu}+C_{\sigma}))}-q\phi\\  $$

### Relaxing assumption 1: accounting for diploid genomes

Simulating diploid GWAS data is important for studying complex genetic traits especially in diploid organisms such as humans. We modify the formulation described in the previous subsection in order to calculate phenotype values that are based on a genotype that could take one of three values: major allele homozygous, minor allele homozygous, and heterozygous.

In many genetic applications, the population would be engineered to only consist of homozygous individuals. However, we choose to account for heterozygous individuals to facilitate the investigation of a wide number of scenarios and genetic traits. We lay out the mathematical procedures needed to calculate the phenotype value for heterozygous individuals using two models of inheritance: 1) the co-dominance model and 2) the complete dominance model. In this section we start by describing the formulation of the co-dominance model before describing the extensions needed for using the complete dominance model. Co-dominance occurs when both alleles in the diploid are fully expressed. That is, the effects of both allele in the diploid are added together to produce the total effect of the genotype of the phenotype’s value.

Assuming the phenotype is affected by a single diploid genetic locus, we modify the phenotype calculation equation (Eq. 1) to account for the genotype state on both chromosomes, as shown in Eq. 11. According to this formulation, the overall genotype state could take one of three values: 0,1,2, corresponding to the allelic states *A*_1_*A*_1_,*A*_1_*A*_2_,*A*_2_*A*_2_ respectively.

11 $$  y =\mu+(g_{1}+g_{2})\alpha+\epsilon, \quad \epsilon \sim N\left(0, (\sigma+(g_{1}+g_{2})\phi)^{2}\right)  $$

Using the same procedure as in the haploid case, we express the parameters in Eq. 11 as follows:

12 $$  \alpha = \sqrt{C_{\mu} V_{Y} / 2pq}  $$

13 $$  \phi = \sqrt{C_{V} V_{Y} / 2pq}  $$

Therefore,

14 $$  \sigma = \sqrt{V_{Y}(1-(C_{\mu}+C_{\sigma}))}-2q\phi\\  $$

Substituting the values of these parameters in Eq. 11 yields the phenotype value for each individual in the population.

### Complete dominance In diploid genomes

An allele shows dominance when it suppresses the effects of the recessive allele. Dominance could exist in three forms: complete dominance, incomplete dominance, and co-dominance. In this section we modify the formulation used in the co-dominance model to account for complete dominance. This type of dominance occurs when the dominant allele completely masks the effects of the recessive allele.

Consequently, the genotype characteristics of this type of diploidy are modified as summarized in Table 2, where *A*_1_ is the dominant major frequency allele and *A*_2_ is the recessive minor frequency allele. The probability of observing a genotype that includes the dominant is denoted by *p*. Similarly *q* is the probability of observing the genotype of two minor frequency alleles. *q* is calculated as the square of the probability of observing a minor allele (i.e., $p_{A2}^{2}$). *α* and *ϕ* are the mean and variance shifts respectively. *g*∈0,1 represents the genotype state, where 0 represents the dominant genotypes (*A*1*A*1,*A*1*A*2) whereas 1 represents the recessive genotype (*A*2*A*2).

**Table 2:** Genotype Characteristics

Genotype	Frequency	Mean	S.D.
*A*_1_*A*_1_,*A*_1_*A*_2_	*p*=1−*q*	*μ*	*σ*
*A*_2_ *A*_2_	$q=p_{A2}^{2}$	*μ*+*α*	*σ*+*ϕ*

Using this formulation, the phenotype could be calculated using the same procedure as the one used for haploid individuals. *α*, *ϕ*, and *σ* can all be calculated using Eqs. (8) to (10). Consequently, the phenotype value could be caluclated using Eq. 1.

### Relaxing assumption 2: accounting for multiple additive loci

In a more realistic scenario, the phenotype value is determined by the genotype state of multiple loci. We modify the phenotype calculation procedure to account for multi-loci effects, assuming an additive model. Linear interaction is only one form of multi-loci interaction. Our phenotype calculation process does not account for nonlinear effects, such as epistasis. However, extending the proposed process to account for these effects is a topic for future investigation.

Extending the phenotype calculation to account for the multiple additive loci is accomplished through modifying Eq. 1 to replace the parameters and variable corresponding to a single loci with vectors of N elements, N being the number of phenotypes affecting loci. The modified phenotype value equation for a haploid genome is presented in Eq. 15

15 $$  y =\mu+\boldsymbol{g^{T}\alpha}+\epsilon, \quad \epsilon \sim N\left(0, \left(\sigma+\boldsymbol{g^{T}\phi}\right)^{2}\right)  $$

According to this formulation, the researcher has to provide two vectors, *C*_*μ*_ and *C*_*V*_ of length N, which represent the mean and variance effect sizes on the total variance for all additive loci. Using these values, and the allele frequencies vector, the parameters of Eq. 15 are calculated as follows:

16 $$  \alpha_{i} = \sqrt{C_{\mu}^{(i)} V_{Y} / p_{i}q_{i}}  $$

17 $$  \phi_{i} = \sqrt{C_{V}^{(i)} V_{Y} / p_{i}q_{i}}  $$

Therefore,

18 $$  \sigma = \sqrt{V_{Y}\left(1-\sum^{A}\left(C_{\mu}^{(i)}+C_{\sigma}^{(i)}\right)\right)}-\boldsymbol{q^{T}\phi}\\  $$

Consequently, the phenotype value of each individual in the population could be calculated through substituting these parameter values into Eq. 15. Similarly, the multi-locus additive phenotype calculation process could be easily extended for diploid individuals through the following modifications, where Eqs. 19 and 15 could be used for the co-dominance and complete dominance cases, respectively. The parameters of Eqs. 19 and 15 are calculated using similar procedures as the ones described in the single loci case.

19 $$  {{\begin{aligned} y =\mu+\boldsymbol{(g_{1}+g_{2})^{T}\alpha}+\epsilon, \quad \epsilon \sim N\left(0, \left(\sigma+\boldsymbol{\left(g_{1}+g_{2}\right)^{T}\phi}\right)^{2}\right) \end{aligned}}}  $$

### vGWAS simulation algorithm

To facilitate the implementation of vGWAS simulators, we translated the mathematical framework into an algorithm, presented in Algorithm 1. The algorithm uses genotype profiles as its input. These genotype profiles could be generated using population simulators or by using real data from genotyping experiments such as the 1000 Genome Project. The algorithm also requires some information about the loci to be simulated, including their number, minor allele frequency, and mean and variance effect size. The algorithm searches the inputted genotypes for loci that meet the specified criteria and then exploits the described mathematical framework to calculate the phenotype value. This algorithm is able to induce both mean and variance discrepancies based on one or more loci. The algorithm accounts for both haploid and diploid genotypes under co-dominance or complete dominance. The algorithm uses Eqs. 15 and 19 to calculate the phenotype value of the haploid genotypes and co-dominant diploid genotypes respectively. The algorithm also uses Eq. 15 to calculate the phenotype value in the case of complete dominance for diploid genotypes.



## Results and discussion

In this section we describe the evaluation of our simulator through the generation of different genetic control scenarios. We conducted two sets of experiments; in the first set, we generated and visualized all the modes of variation supported by our tool to demonstrate its ability to mimic realistic patterns. In the second set of experiments, we used common GWAS and vGWAS approaches to recover the loci generated using our algorithm. Finally, we highlighted several applications in which our simulation process could be useful.

The simulation algorithm was implemented as Python scripts and has been tested on a Unix platform. The simulation scripts were used to calculate quantitative phenotypes from genotype profiles generated using ms [16] and GENOME [18] which are two popular coalescent genotype simulators. The simulation scripts use Eq. 15 and Eq. 19 to calculate the phenotype value of the haploid genotypes and complete-dominance diploid genotypes respectively. The simulator also uses Eq. 15 to calculate the phenotype value in the case of co-dominance for diploid genotypes. In our implementation, we utilized scripts from [23] for performing basic tasks such parsing genotypes and writing the output files.

### Phenotype distribution

Our first experiment aims to verify that the values of the generated phenotype are in accordance with the desired distribution. We generated a segment containing 10,000 genotypes for 2,000 samples using Hudson et al.’s ms [16] simulator. From the generated genotypes, we generated six sets of results corresponding to two modes of ploidy, each containing three types of effects. We used our simulation algorithm to calculate phenotype values for haploid genotypes, diploid genotypes under co-dominance. For each mode of ploidy, we generated created three sets of phenotype values corresponding to three types of loci. We created one locus in each of these sets. In the first set, the locus had a mean effect size of 5% and no variance effects. The second locus had no mean effects, and a variance effect size of 5%. The third set, the single locus had both mean and variance effects, each with a 5% effect size.

**Fig. 1:**
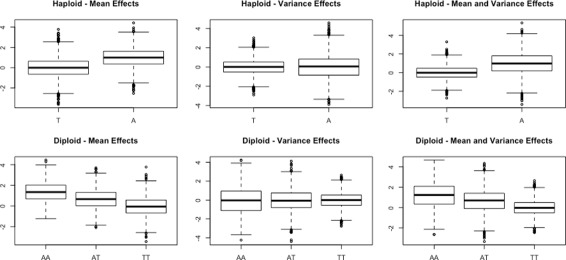
Phenotype distribution under different genotype effects. The columns represent the three types of simulated genotype effects from one QTL with an effect size of 5%. The rows represent the two modes of ploidy our model accounts for

We visualized the phenotype values of these six sets of results using box plots, as shown in Fig. 1. All the results follow a normal distribution. The base-line phenotype mean value was set to zero in all simulations, and was observed accordingly in the results. The first column of Fig. 1 shows the mean effects of a locus on the two modes of the simulated ploidy. It is observed that these effects are causing a shift in the mean phenotype value without having any noticeable effects on the variance in the two plots of that column. In the second row, co-dominance produced three equidistant mean levels for the phenotype. In the second column, instead of causing mean shifts, the locus caused different variance shifts across the ploidy scenarios. Finally, the locus in the third column had both mean and variance effects which translated to coinciding mean and variance shifts in the phenotype values. As a conclusion, our vGWAS simulations produces phenotype values that are in accordance with the desired and intended behavior.

### Association signal recovery

To demonstrate the utility of our algorithm, we performed a second experiment which involved simulating genotypes based on realistic settings and then using common GWAS and vGWAS analysis tests to recover the simulated loci. The purpose of this experiment is to show that the GWAS and vGWAS signals simulated using our algorithm could be retrieved using common GWAS/vGWAS detection algorithms. To that purpose we used the GENOME [18] simulator to generate genotypes with similar characteristics as those observed in the arabidopsis thaliana 120Mbp genome [24–26]. We generated three diploid chromosomes with 50,000 SNPs each for a population size of 10,000 with a population recombination parameter of r = 8*X*10^−^3. We then used our simulation algorithm to generate three loci, one on each chromosome. The first locus has a mean effect size of 5% and no variance effects. Similarly, the second locus has a variance effect size of 5% and no mean effects. The third locus has a total effect size of 6%, divided equally between mean and variance effects.

To recover the simulated signals, we used three methods:

1. We used Plink’s ‘assoc’ function which is a GWAS detection method. This method attempts to fit a linear regression at each genotype to determine if there is a relation between the varying genotype values and the corresponding phenotypes. Eq 1. This approach mainly attempts to estimate the mean shift sizes occurring at the different genotype states in the presence of a normally distributed residual having a constant variance term.
2. The Brown-Forsythe test which is a vGWAS detection method. This method single-pass test which measures the significance of the variance separation observed at one tested genotype at a time and ultimately produces a p-value statistic for that genotype. This test has been commonly used to check for variance heterogeneity in vGWAS experiments. We implemented this test using R’s Levene Test package [27].
3. We implemented the DGLM association method described in [9] which is both a GWAS and vGWAS method. This method attempts to recover both mean and variance shift-causing loci from the data simultaneously.

**Fig. 2:**
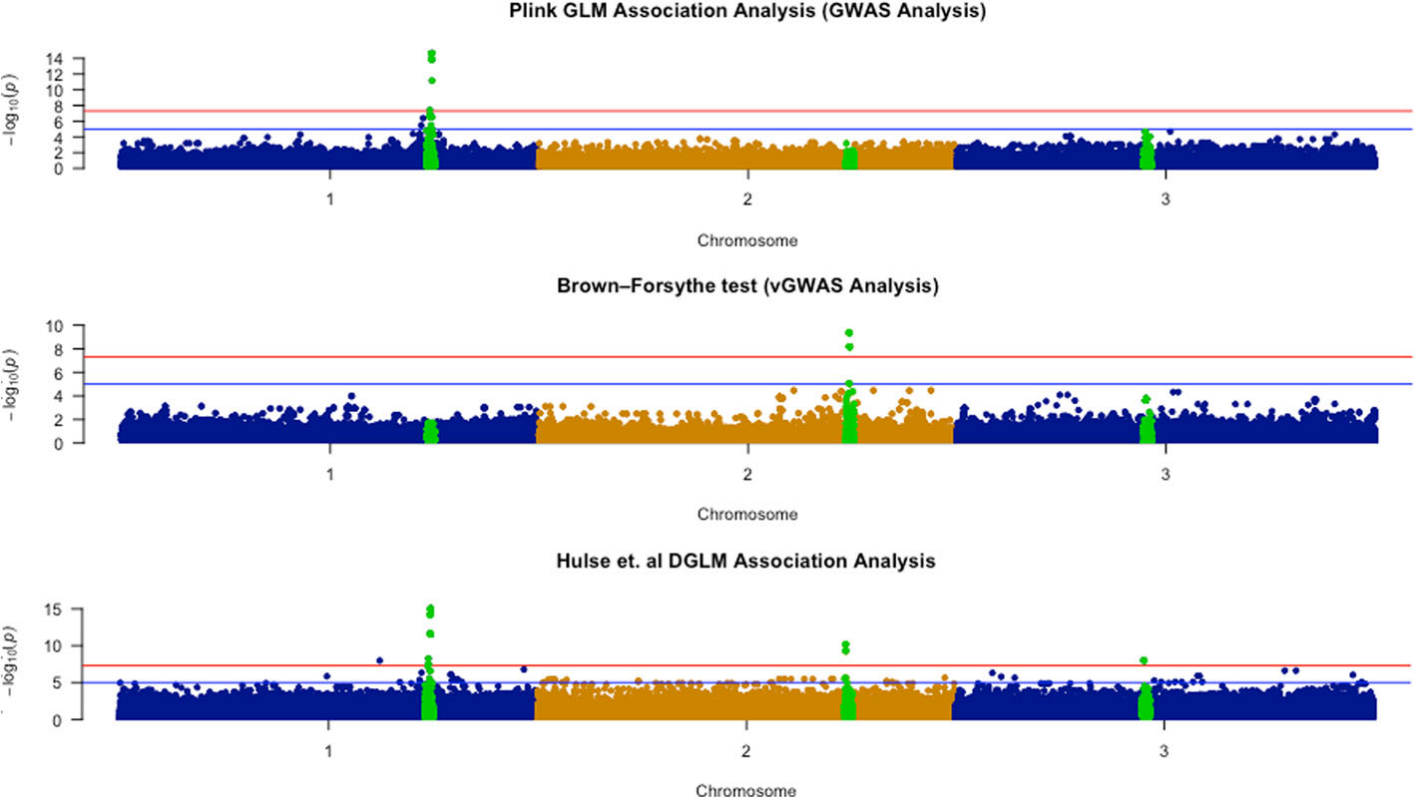
Association signal recovery using the common GWAS and vGWAS analysis algorithms. GWAS analysis was carried out using Plink 1.9. vGWAS analysis was carried out using the Brown-Forsythe R package [27]. In addition, the DGLM analysis method proposed in [9] was implemented and tested in the third panel of the figure

Figure 2 present the results of the three tests performed. The first and third panels in the figure show that both the Plink association test and the DGLM method were able to detect the GWAS locus on the first chromosome. This result is expected since both Plink and Hulse et al.’s DGLM method are capable of detecting pure GWAS signals. On the other hand, and since the Brown-Forsythe test only checks for variance shifts, it was not able to detect that locus. On chromosome two however, the Brown-Forsythe test and the DGLM method were both able to detect the vGWAS locus, since they are both designed to detect significant variance shifts. This locus has gone unnoticed by the Plink association method which does not take variance effects into account. Lastly, the third locus which has both mean and variance effects exhibited only a mild significance in the first two tests and therefore it could potentially go undetected in typical settings. In contrast to that, the Hulse et al. DGLM method was able to detect a more significant effect for this locus. Each of the simulated GWAS and vGWAS signals account for 3% of the total variance. Taken separately, each of these signal has a modest contribution to the total variance. However if the GWAS and vGWAS signals at the locus are combined together, they will exhibit a substantial significance. Since Hulse et al.’s DGLM method combines both GWAS and vGWAS signals in its detection process, it was able to detect a clearer signal for the GWAS/vGWAS locus on the third chromosome. This result also highlights the advantage vGWAS methods that are able to account for combined mean and variance effects.

### Simulation scenarios

**Fig. 3:**
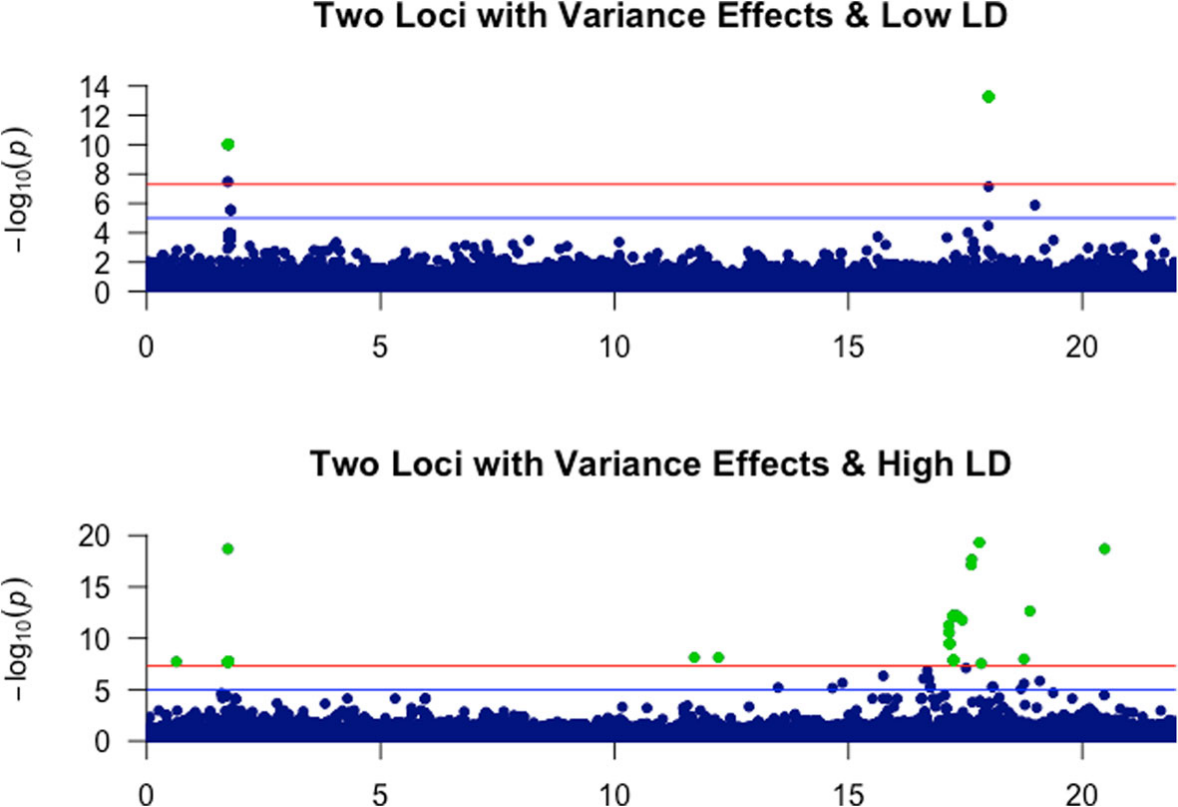
Association signal of two loci with variance effects under different linkage disequilibirium conditions. The recovery was performed using the Brown-Forsythe R package [27]

To further demonstrate the utility of our simulation algorithm, we developed several scenarios to show how simulation could help unveil important evolutionary and structural information in the genome. In the first experiment, we used GENOME [18] to generate two 25Mb segments containing 50,000 SNPs each. We varied the recombination rate between the two segments in order to create two different linkage disequilibrium (LD) conditions: a low LD segment, and a high LD segment. In each of these segments, we simulated two loci with 7% variance effects and no mean effects. We then visualized the p-values of these loci in Fig. 3. As could be seen from the figure, when LD was low, the effects of the two loci were confined to the region in which they occur. There were no association signals detected in other regions of the genomic segment. However, this is not the case in the high LD scenario where the association signal was more dispersed around the affected loci. LD generally decreases as the distance from the affected locus increases. In the low LD scenario, the region affected by LD decreases more steeply than in the high LD case. Consistant with realistic genomic structure, some regions might have a stronger LD level with an affected locus than their neighboring regions. Simulation could help identify those regions as it did in the second panel of Fig. 3. The panel shows two significant vGWAS signals at around 12Mb although no vGWAS loci were simulated at these locations. These genomic locations exhibited a significant vGWAS signal due to their LD connection with one or both simulated vGWAS loci.

**Fig. 4:**
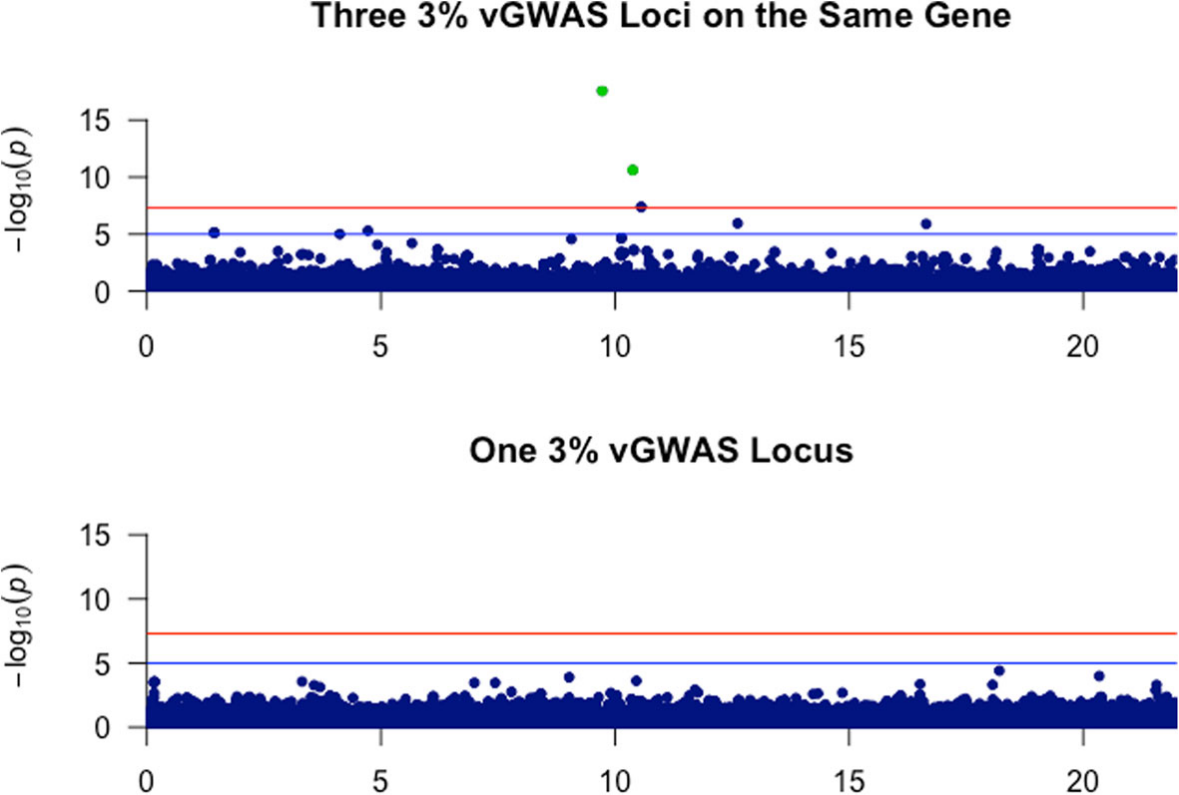
Association signal of three loci with variance effects in the same gene. The recovery was performed using the Brown-Forsythe R package [27]

In the second experiment, we used GENOME to generate two 25Mb segments containing 50,000 SNPs each. In the first segment, we simulated three loci each having a 3% variance effect and no mean effects. We placed the loci within 2000 bases of each other to resemble loci occurring in the same gene. In the second segment, we simulated only one locus having a 3% variance effect and no mean effects located in the same region as loci of the first segment. We present the p-value patterns of this experiment in Fig. 4. The main observation that could be made from the first panel of the figure is that although each locus individually has a low variance contribution, these loci exhibited a significant vGWAS signal. This highlights the effect of LD on vGWAS loci when they occur in close proximity in the genome. In such a scenario, the vGWAS loci that are in LD witness additional variance contributions from the nearby vGWAS loci. In contrast, the vGWAS locus of the second panel, which only contains a single vGWAS loci with 3% effects did not exhibit a significant vGWAS signal. It is worth mentioning that we placed the locus of the second panel at the location of each of the loci of the first panel and observed similar results at all three locations.

## Conclusion

In this work we presented a mathematical framework for simulating quantitative vGWAS data. We translated the mathematical framework into a simulation algorithm, which, to the best of our knowledge, is the first vGWAS simulation algorithm in the literature. The presented algorithm generates quantitative vGWAS phenotype values using genotype profiles from common population simulators. The algorithm could be applied to either haploid or diploid genotypes with different modes of dominance while incorporating the effects of multiple GWAS and vGWAS loci using an additive model. To demonstrate the utility of our algorithm, we used common GWAS and vGWAS analysis methods to detect the loci generated using our algorithm. We hope that the presented work will motivate the further evaluation of current vGWAS methods and drive the development of new methods to address the limitations of current methods.
